# Heartbeat and Economic Decisions: Observing Mental Stress among Proposers and Responders in the Ultimatum Bargaining Game

**DOI:** 10.1371/journal.pone.0108218

**Published:** 2014-09-23

**Authors:** Uwe Dulleck, Markus Schaffner, Benno Torgler

**Affiliations:** 1 Queensland Behavioural Economics Group (QuBE), School of Economics and Finance, Queensland University of Technology, Brisbane, Australia; 2 CREMA – Center for Research in Economics, Management and the Arts, Basel, Switzerland; 3 EBS Universität für Wirtschaft und Recht, EBS Business School, Oestrich-Winkel, Germany; Institutes for Behavior Resources and Johns Hopkins University School of Medicine, United States of America

## Abstract

The ultimatum bargaining game (UBG), a widely used method in experimental economics, clearly demonstrates that motives other than pure monetary reward play a role in human economic decision making. In this study, we explore the behaviour and physiological reactions of both responders and proposers in an ultimatum bargaining game using heart rate variability (HRV), a small and nonintrusive technology that allows observation of both sides of an interaction in a normal experimental economics laboratory environment. We find that low offers by a proposer cause signs of mental stress in both the proposer and the responder; that is, both exhibit high ratios of low to high frequency activity in the HRV spectrum.

## Introduction

*I hesitate to say that men will ever have the means of measuring directly the feelings of the human heart. A unit of pleasure or of pain is difficult even to conceive; but it is the amount of these feelings which is continually prompting us to buying and selling, borrowing and lending, labouring and resting, producing and consuming; … but, just as we measure gravity by its effects in the motion of a pendulum, so we may estimate the equality or inequality of feelings by the decisions of the human mind.*

William Stanley Jevons (1871), Introduction to *The Theory of Political Economy*

Although laboratory experiments in economics are sometimes criticised for straying too far from the field, they still offer the comparative advantage of unlocking the ‘black boxes’ of human decision making under strictly controlled conditions. One prominent example is the ultimatum bargaining game (UBG) [1], which clearly demonstrates the role of social, or other-regarding, preferences in individual decisions. In addition to being used in a large number of behavioural studies, the UBG has also been employed in neuroeconomic investigations, which sometimes rely on the highly demanding brain scanning technologies used in neuroscientific research [2]–[4]. These technologies, however, seriously constrain the experimental laboratory setting; in particular, by limiting the number of possible participants and restricting social interactions between them. In this complementary study, therefore, we strive to overcome such technological limitations by employing the simpler physiological measure of heart rate variability (HRV).

HRV, defined as a variation in the time interval between heart beats, has been theoretically and empirically shown to *indirectly* index regulated emotional responses (for a discussion, see [5]) by registering a ‘cardiac signature’ or ‘theatre’ of emotions. This ability, however, comes at the cost of having only an indirect measure of neural processes. In addition, as an index of regulated emotional responding, HRV is unable to capture the multifaceted processes of emotions. Empirically, therefore, we are employing an index of sympathovagal balance that serves as an *indirect* indicator of mental or psychic stress. At the same time, the controlled laboratory setting allows us to limit participants' movements (e.g., standing up, walking around), thereby reducing as much as possible any other physiological ‘noise’ that could interfere with the effect under exploration.

Specifically, we run standard economic laboratory experiments using heart rate monitors the size of a 2005 IPod (Figure S1 in File S1), which, by recording a participant's electrocardiogram (ECG) with medical levels of accuracy, provide a measure of HRV. Because cardiac automaticity is under the control of the autonomic nervous system (ANS), this measure provides indirect information about the subjects' stress levels (emotional responses) [6]. More explicitly, because the heart rate measures the body's physical reactions to stressors, its variability provides insights into the ANS itself; most especially, the balance between sympathetic (fight and flight) and vagal (rest and relax) activity [7]. HRV data are also empirically linked to individual anxiety, emotional personality, and the activity of several brain areas [8], meaning that HRV provides insights into both mental activities and emotions. Not surprisingly, therefore, the use of this measure in economics research is increasing, especially given the finding that HRV is affected by payoff-relevant feedback [9]–[11].

Nevertheless, until recently neuroscience avoided exploring the complex aspects of moral emotions or moral values, other than providing systematic evidence on pride and embarassement from cases of frontal lobe damage [12], [13] and showing that violations of moral norms can evoke moral revulsion or disgust [14]. In general, brain scanning technologies help to identify moral and other motives that drive human behaviour by indicating areas of the brain that are activated during decision making [15]. Unfortunately, however, brain scanning restricts the type of interactions that can be studied – in particular, the interactive element in standard economic experiments – thereby preventing simultaneous exploration of the behaviour of all parties involved.

For our study, we use a standard UBG in which two players are instructed to share a certain amount of money. The first player – the proposer – offers a way to split the amount, while the other player – the responder – can choose to accept this split (giving both participants the same amount) or reject this offer, in which case neither player receives anything. We find that the modal and median ultimatum offers are around 40–50 percent while the means are between 30 and 40 percent. As in other studies [16], offers below around 20 percent are rejected about 50 percent of the time, while, offers in the range of 0 to 10 percent or above 50 percent are few.

Not only is the UBG one of the most heavily studied games in experimental economics, it is also strongly tied to social preferences [17]–[19] and the analysis of emotions in general. Above all, as Handgraaf, Van Dijk and De Cremer aptly point out, the game mirrors reality: ‘Ultimatums are everywhere. A woman in the train who tells her child to turn down the volume of a gameboy “or else…”; a police officer who tells a drunk driver to walk home if he wants to avoid his license being withdrawn – they are all instances of ultimatums’ ([20] p. 263). In fact, ultimatum games clearly model social-problem decisions, which ‘can be non-stationary in a very specific sense: the value associated with one agent's action depends criticially on the changing actions (and mental states) of other social agents’ ([21] p. 159).

This present study focuses on emotional determinants of decision making in ultimatum situations, an issue often addressed using interview or survey based research. Pillutla and Murnighan [22], for example, use open-ended questions to examine purchase negotiations, while Bosman, Sonnemans, and Zeelenberg [23] survey ultimatum game responders on the intensity of each element in a list of nine emotions. Both studies find a significant negative relation between the offer and the intensity of negative emotions, as well as a positive relation between the offer and the intensity of positive emotions. They also discover that a responder's expectation of the offer is positively related to the intensity of negative emotions, a finding that can be reconciled with the results in the neuroeconomic literature [2]. In a study that complements UBG-based neuroeconomic research, van 't Wout et al. [24] overcome the bulkiness of brain scanners by using skin conductance; however, as in the neuroeconomic literature, their focus on responder behaviour neglects the proposer.

We are in fact aware of only two studies [25]–[26] that explore the emotional responses of proposers in a UBG. In the first, Ketelaar and Au [25] use a repeated ultimatum game to elicit self-reported feelings of guilt, asking participants to pair up with a class colleague and adopt fixed proposer and reponder roles for two rounds, the second of which takes place a week later. The study results indicate that guilty reactions to selfish offers in the first round increase the proportion of generous offers a week later. The second analysis, by Nelissen et al. [26], links fear to the concern that low offers might be rejected (risk of rejection) and sees guilt as taking into account the concerns and interest of others. Nelissen et al. measure anticipated fear based on the extent to which participants felt “afraid”, “worried” or “nervous” about their offer being rejected if it were less, and guilt based on the extent to which they felt “guilty” or “bad about what they did” or “regretted their decisions” ([26] p. 41). These authors induce guilt by asking subjects to either report on a recent event in their lives that made them feel guilty (treatment) or describe an ordinary neutral event (control). Their results indicate that the treatment group makes higher offers than the control group.

In line with this finding, in our study, the HRV measure provides clear evidence that low offers in the UBG lead to high levels of physiological arousal in responders *and* proposers. We interpret the arousal as a sign of mental stress. Using our HRV technique, therefore, allows us to combine *behavioural* evidence with proposers' physiological reactions when making unfair offers. We provide a more detailed discussion of the current evidence on HRV use in laboratory experiments exploring human interactions in the next section prior to describing our experiment, reporting the results, and outlining our conclusions.

## HRV in Laboratory Experiments on Human Interactions

The HRV measurement method records information on activity in two major parts of the ANS, the sympathetic and parasympathetic systems (for a discussion, see [27]). More specifically, because the oscillations in heart rate generated by these two branches of the ANS occur at different speeds or frequencies [5], the timing difference can be used to identify the extent of both activities. Activity in the sympathetic system is reflected in the low frequency band (LF [0.033–0.15 Hz]) and that in the parasympathetic system by high spectral power in the high frequency band (HF [0.15–0.4 Hz]) (see also [28]). That is, individuals react to mental stress with either increased sympathetic and/or decreased parasympathetic activity [29]. Hence, the ratio of activity in the low frequency band to that in the high frequency band (i.e., the LF/HF ratio) can be used as an index of sympathovagal balance [5], which serves as a useful indicator of psychic stress. The LF/HF ratio is obtained by applying a standard smoothed pseudo Wigner-Ville distribution (SPWVD) transformation using the cubic interpolated heart rate signal (5 Hz) and 512 frequency bins. Based on the context in which our study takes place, we interpret the physiological arousal captured by HRV as a measure of mental or psychic stress. We do not provide insights into the exact physiological and psychological mechanism; while the topic is worthy of further investigation, it is outside the scope of this study.

Most important for our study is that HRV measures are a valuable tool for understanding responses to social interactions or human performance. Falk et al. [30], for instance, observe that unfair pay is correlated with lower heart rate variability (higher stress), while Dulleck et al. [8] show that in a UBG setting, stress levels during a communication stage are correlated with the size of the offer. Dulleck et al. [27] also use an HRV measure that captures psychic costs from the contemplation of real or imagined actions (tax evasion) to assess the psychic cost of breaking social norms (tax compliance). Their results provide empirical evidence of a positive correlation between tax compliance and psychic stress, a finding consistent with empirical evidence that in a situation of conflict between self-interest and group interest (social dilemma), moral emotions help individuals solve the social dilemma situation [31]. Brandts and Garofalo [32], on the other hand, report being unable to correlate HRV with success in the resolution of a task, while Van Lange et al. [33] show that HRV measures can seemingly influence the experimental outcome by promoting behavioural trust in trust games and reciprocal giving in the trustee. They point to interpersonal (touch and communications of care) and intrapersonal mechanisms (arousal and self-awareness) as possible explanations for this finding.

## Experimental Design

Our laboratory experiment was approved by the QUT University Human Research Ethics Committee (UHREC, ethicscontact@qut.edu.au). The study also involved written informed consent by participants, a procedure approved by the ethics committee. The experiment was conducted at the Queensland University of Technology over a three-week period in May 2008. The 156 voluntary participants were recruited primarily from a first-year economics course using a faculty wide invitation email and in-lecture advertisement. Those recruited were informed that the experiment would include measurement of their heart rate and that they would not be allowed to eat or drink anything except water 90 minutes prior to the experimental session. The entire study comprised a total of 13 sessions conducted with six proposers and six responders. All sessions were held in the afternoon to minimise the effect of daytime variation in the heart rate, and none of the participants reported previous experience with such an experiment in the follow-up questionnaire.

The participants were randomly divided into two groups (proposers and responders) and invited to different locations to avoid potential biases from previous social interactions between groups. After being welcomed to the experiment, they received instructions about and assistance with applying the heart rate monitors, which involved placing three single-use electrodes connected to a Holter Medilog AR4 heart rate monitor on the participant's chest. The participants then had to climb two sets of stairs to reach the computer lab, a controlled physical activity designed to generate a baseline measurement for the individual HRV components.

The computer-based experiment was programmed and conducted using z-Tree [34]. Participants were presented with the instructions for the ultimatum bargaining game on the screen as they sat in front of the computer (Figure S2 in File S1) and also heard the instructions read by a native English speaker. This technique served two purposes: it ensured that all participants (1) heard the instructions and (2) knew that the same rules applied to all experimental subjects. Before the experiment began, participants also had to answer two control questions to check their understanding of the instructions (see Figure S3 and S4 in File S1).

In our experiment, the UBG consists of nine rounds in which each proposer is randomly matched with one responder in every round (see Figure S5 in File S1 and Figure S6 in File S1). Each proposer receives 360 cents (Australian $) to split with a responder, who must then decide to accept or reject the suggested division; if the offer is rejected, neither player receives payment. At the end of each round, both participants receive a statement of all the information on this round and their individual payoffs (see Figure S7 in File S1). In 10 out of the 13 sessions conducted, the participants received the aggregated payoff from all nine rounds as well as a show-up fee of $5. In the remaining three sessions, participants received a lump sum payment of $17 for participating in the ultimatum bargaining part of the experiment. The UBG itself is followed by a risk-aversion elicitation activity (see Figure S8 in File S1).

The experiment also includes a communication phase in which proposers and responders meet to talk. Specifically, two proposers are grouped together with two responders and given the chance to talk freely without discussing the experiment. This communication phase was implemented after the sixth round in 10 of the sessions and at the end of the ninth round in the other 3 sessions. This manipulation, which is designed to increase the feeling of closeness with other participants, tests for an ad-hoc reference group.

The descriptive statistics for the experimental data are summarised in Table 1, which lists the average offer in cents, the average offer as a percentage, the acceptance rate, the average accepted offer as a percentage, the average declined offer as a percentage, and the gender and age distribution for various subgroups. These subgroups are based on basic socio-demographic data and other control variables collected by questionnaire at the end of the experiment. Overall, the findings are in line with the results from other UBG-based research (e.g. [35]).

**Table 1:** Summary of the experimental data.

Group	N	Offer	Offer	Accept	Accept	Decl.	Gender	Age
		cents	%	%	offer %	offer %	% female	years
All	156	152.02	42.23	71.30	45.99	33.02	42.95	21.14
		(28.21)	(7.84)	(21.08)	(9.19)	(11.86)		(4.42)
Proposer	78	152.05	42.23				42.31	21.54
		(36.73)	(10.20)					(4.52)
Responder	78			71.31	46.90	31.45	43.59	20.74
				(24.75)	(7.14)	(10.80)		(4.32)
Female	67	152.77	42.11	73.98	46.06	29.20	100.00	22.04
		(32.00)	(5.06)	(24.75)	(5.07)	(11.74)		(5.61)
Male	89	151.51	42.31	69.24	47.54	33.16	0.00	20.46
		(40.19)	(3.91)	(24.84)	(8.38)	(9.86)		(3.12)

**Note**: Standard deviations in brackets.

## Data Analysis and Results

To analyse the participants' behaviour, we look in detail at two distinct events during the experiment: (1) the point in time at which the proposer has locked in an offer and is awaiting the responder's decision and (2) the responder's decision in reaction to that offer. As key decision variables, we focus on the size of the proposer's offer (in % of total distributed payment) and the responder's decision to accept or reject (1 = accept, 0 = reject). Our stress indicator, which is based on the smoothed pseudo Wigner-Ville distribution (SPWVD) wavelet transformed time-frequency distribution, is the ratio of integrated power in the low frequency band (LF [0.033–0.15 Hz]) to that in the high frequency band (HF [0.15–0.4 Hz]). We test the fit of our measure using an alternative time-frequency distribution estimation method, but our results remain largely robust. The resulting HRV measure is then averaged over certain time intervals starting at the moment a decision is registered in the experimental software. The data used in this study are available on the webpage of the Queensland Behavioural Economics Group.

### Data Adjustments

Data adjustment is necessary because the time data of the events in a computer-based experiment is prone to variation caused by network traffic issues [34]. As a result, the time recorded gives only an indication of when the event actually took place; that is, when the information screen was in fact visible to the participant. The network setup used in our experiment initially caused a delay of up to three seconds, but we resolved this issue in subsequent experiments by placing the lab computers on their own private subnet detached from the university network [27]. We must also assume a lag between the time a participant makes a decision and the point at which the decision is entered into the computer. To circumvent both problems, we allow for a variation of at least five seconds in the time observation of any event. More specifically, in identifying the strength of a potential effect, we use averaged data over different time intervals – (0,10), (0,20), (0,30) and (0,40) seconds after the time a participant was asked to make the decision – in order to capture any reasonable time lag and/or inaccuracies in the time recording.

Finally, we must account for individual differences in the strength of the effect and the underlying signal. First, we address participant heterogeneity by using an individual fixed effects specification equivalent to a dummy variable for each individual. Second, we use an innovative outlier detection routine – in the spirit of Hendry and Krolzig's [36] impulse-saturation method – which allows inclusion of additional individual characteristics while still controlling for individual effects. The routine repeats the analysis using randomly selected blocks of individual dummy variables, retaining those that show a significant influence in multiple repetitions. Thus, in addition to controlling for personal characteristics such as gender, health status, or risk attitude, to capture further individual effects, we also introduce the LF/HF ratio recorded during the walking phase (climbing two sets of stairs) at the beginning of the experiment to control for different baseline levels in the signal.

### Models and Estimation Tables

#### 1. Proposer

To gauge the effect of the economic decision-making process on mental stress levels, we first look at the reaction of proposer *i* when tendering an offer in period *t*. To do so, we estimate a model that postulates a linear relation between the level of the stress indicator (LF/HF ratio) as dependent variable and the size of the offer (as a percent of the total redistribution sum in each round). This model includes control variables that account for differences in the experimental setup (such as pre- or post-communication phase or whether or not it was a paid session); a time/period variable (number of previous rounds); and a subject dummy to control for individual effects as independent variables. In the specification without individual dummies, the control variables also include individual characteristics like gender, health (whether the participant takes any medication; 1 = yes, 0 otherwise), and the baseline LF/HF ratio as measured during the stair climbing phase. We also use different time intervals [t0,t1] of means (10, 20, 30, 40 seconds) when measuring the level of the stress indicator (LF/HF ratio) after the decision event in question.

Table 2, which reports our four estimations using the different time intervals for the two techniques, shows a statistically significant relation between the proposer's offer and his or her stress level. The sign of the coefficient is negative, which indicates that making a higher offer (one more likely to be accepted) reduces the level of stress (i.e., decreases sympathetic and/or increases parasympathetic activity). Making a low offer, in contrast, increases the proposer's stress level. It is also clear that the coefficient increases and decreases in size and significance over the time interval shown, with the (0–20) second interval exhibiting the strongest effect. It should be noted, however, that 12 out of the 78 recordings for the proposers (and 14 for the responders) were discarded because of irregularities in the ECG recording, caused mostly probably by dislocated electrodes. Nevertheless, as shown in the supporting information (Table S1 in File S1), the behaviour of the excluded participants does not differ from that of the rest of the sample, lending validity to the data analysis without those observations.

**Table 2:** Proposer's post-offer stress level.

	FE				OLS			
	(0–10)	(0–20)	(0–30)	(0–40)	(0–10)	(0–20)	(0–30)	(0–40)
Offer	−1.951**	−2.468***	−1.739**	−1.076	−1.288	−1.891**	−1.29**	−0.93
	(−2.00)	(−2.83)	(−2.17)	(−1.40)	(−1.54)	(−2.49)	(−2.10)	(−1.57)
Communication	0.168	−0.377	−0.608	−0.711*	0.113	−0.505	−0.671	−0.634
	(0.31)	(−0.79)	(−1.38)	(−1.68)	(0.25)	(−1.21)	(−1.77)	(−1.74)
Paid session					0.023	0.221	0.288	0.31
					(0.08)	(0.82)	(1.2)	(1.33)
Age					−0.012	0.03	0.029	0.046**
					(−0.39)	(1.33)	(1.41)	(2.10)
Gender (female)					−0.657**	−0.905***	−0.616***	−0.627***
					(−2.56)	(−3.78)	(−2.81)	(−2.92)
Health					−0.38	−0.199	−0.438	−0.4
					(−1.25)	(−0.67)	(−1.62)	(−1.52)
Baseline					0.108***	0.088**	0.132***	0.130***
					(3.08)	(2.35)	(4.61)	(4.72)
PERIOD	Yes	Yes	Yes	Yes	Yes	Yes	Yes	Yes
INDIVIDUAL	Yes	Yes	Yes	Yes	Yes	Yes	Yes	Yes
*R* ^2^	0.02	0.04	0.04	0.06	0.23	0.29	0.31	0.36
F	1.286	2.146	2.402	3.083	5.863	7.512	9.634	10.916
*Pr*(*F*>*f*_0_)	0.064	0.000	0.000	0.000	0.000	0.000	0.000	0.000
N	594	594	594	594	594	594	594	594

**Notes**: Dependent variable: average LF/HF ratio over 10, 20, 30 and 40 seconds after the subject puts forward the offer. Significance levels: * = 0.05<*P*<0.10, ** = 0.01<*P*<0.05 and *** = *P*<0.01. *t*-statistics in parentheses.

#### 2. Responder

To examine the reaction of the responder, we introduce into the model a dummy variable indicating acceptance of the offer (*A*). Since a responder's reaction depends on the size of the offer received, the height of the offer (*O*) and the interaction term (*A×O*) can also be included. The interaction term helps us to identify the effect of the acceptance while controlling for different levels of offers. For the responder, we estimate a linear relationship between the level of the stress indicator (LF/HF ratio) as dependent variable and the acceptance decision, the offer, the cross effect of both and the control variables, a period variable, and an individual effect dummy.

In Table 3, which reports the regression results using the same time intervals as in Table 2, the variables of interest are again highly statistically significant, the signs are in the expected direction, and again the (0–20) second interval exhibits the strongest impact. In fact, the result for this interval in the fixed effects specification clearly shows that the pure effect of accepting an offer of zero share is positive (2.616–6.769*0 = 2.616), indicating that the acceptance of such an offer produces a significant increase in stress level. On the other hand, the effect of accepting an equal offer of a share of 50 percent of the total amount to be divided (2.616–6.769*0.5 = −0.768) is slightly negative, indicating that accepting a 50 percent offer somewhat reduces the responder's stress level. Rejecting a higher offer, however, also induces stress, suggesting that the responder's enforcement of the norm carries psychic costs. Taking this interpretation one step further, we can calculate the mean cut-off value at which the acceptance of such an offer no longer causes increased stress; namely, at (2.616–6.769*x = 0→*x* = 0.386) or an offer of around a 40 percent share of the amount to be divided.

**Table 3:** Responder's stress level after acceptance or rejection.

	FE				OLS			
	(0–10)	(0–20)	(0–30)	(0–40)	(0–10)	(0–20)	(0–30)	(0–40)
Accept (*A*)	1.703**	2.616***	2.409***	1.819**	1.643**	2.45***	2.271***	1.898**
	(2.05)	(3.08)	(2.84)	(2.31)	(2.11)	(3.07)	(2.85)	(2.58)
Offer (*O*)	4.168**	7.312***	6.28***	4.211**	4.406**	7.532***	6.269***	4.507***
	(2.30)	(3.94)	(3.38)	(2.45)	(2.54)	(4.22)	(3.54)	(2.75)
Interaction (*A×O*)	−4.129**	−6.769***	−5.948***	−4.047**	−4.33**	−6.962***	−6.055***	−4.527**
	(−1.98)	(−3.18)	(−2.79)	(−2.05)	(−2.18)	(−3.41)	(−2.98)	(−2.41)
Communication	−0.655	−0.834	−1.165*	−1.255**	−0.908*	−0.931*	−1.246**	−1.166**
	(−1.10)	(−1.37)	(−1.91)	(−2.22)	(−1.71)	(−1.70)	(−2.30)	(−2.32)
Paid session					0.962**	−0.602	−0.339	−0.172
					(2.45)	(−1.52)	(−0.85)	(−0.47)
Age					0.034	0.052	0.019	0.029
					(0.95)	(1.39)	(0.52)	(0.83)
Gender (female)					−0.655**	−2.04***	−1.177***	−1.435***
					(−2.17)	(−6.30)	(−3.76)	(−4.59)
Health	No	No	No	No	Yes	Yes	Yes	Yes
Baseline	No	No	No	No	Yes	Yes	Yes	Yes
PERIOD	Yes	Yes	Yes	Yes	Yes	Yes	Yes	Yes
INDIVIDUAL	Yes	Yes	Yes	Yes	Yes	Yes	Yes	Yes
*R* ^2^	0.04	0.09	0.13	0.15	0.33	0.36	0.40	0.45
F	1.848	3.994	6.119	7.474	7.50	8.14	9.765	11.025
*Pr*(*F*>*f*_0_)	0.000	0.000	0.000	0.000	0.000	0.000	0.000	0.000
N	585	585	585	585	585	585	585	585

**Notes**: Dependent variable: average LF/HF ratio over 10, 20, 30 and 40 seconds after the subject puts forward the offer. Significance levels: * = 0.05<*P*<0.10, ** = 0.01<*P*<0.05 and *** = *P*<0.01. *t*-statistics in parentheses.

Overall, the results reveal that both the proposer's and responder's stress levels are affected by the decisions made during the UBG. They also indicate that, on average, accepting offers that deviate below the 40 percent level causes higher stress levels in responders.

### Extension

To extend the analysis, we look for evidence that these physiological reactions also have behavioural consequences; evidence that is hard to come by given that a fully self-aware participant would anticipate the stressful experience and adapt his or her behaviour accordingly. The fact that we can find residual evidence for stressful events in the analysis above, however, indicates that our participants were not fully able to anticipate and incorporate their emotions. This observation thus raises the question of whether the stress level associated with the decision in the previous period (*t*−1) affects behaviour in the current period. Unfortunately, directly analysing this effect by relating the stress level of the previous period to the behaviour in the current period is not fruitful for either the level of the proposer's offer or the responder's decision to accept or reject. That is, even apart from the adaptation issues already mentioned, the statistical analysis is further hindered by the lack of a proper control in the model for individual heterogeneity in the HRV. As an alternative, we resort to findings from a difference approach whereby the linear relation is estimated using the change in the LF/HF ratio as dependent variable and the change in offer size and control and independent variables.

Here, the objective is to explore how the difference in the level of offers made (Δ*O_i,_*_(*t-*1,*t*)_) by proposer *i* between the two periods *t*−1 and *t* relates to the differences in the stress level (Δ*H_i,_*_(*t-*1,*t*)_) as measured over a 20 second interval after the decision in both periods. Our hypothesis is that participants can trade off a higher stress level for a higher offer if it affects them negatively.

The results in Table 4 show that there is indeed a negative relation between the difference in the offer and the difference in the stress level. For these estimates, we report four different specifications, one using a fixed effects model to account for individual heterogeneity (1), and three that show results from a standard OLS regression with added structural and individual control variables (2–4). Although the impact of the additional control variables is minimal, in the last specification, it is clear that the overall validity of the model suffers.

**Table 4:** Proposer's stress level (differences approach).

	(1)	(2)	(3)	(4)
	(FE)	(OLS)	(OLS)	(OLS)
Diff. Offer (Δ*O*)	−2.737***	−2.768***	−2.681***	−2.684***
	(−2.92)	(−3.16)	(−3.07)	(−3.06)
Communication			−0.336	−0.331
			(−0.62)	(−0.61)
Paid session			0.239	0.228
			(0.70)	(0.66)
Age				−0.008
				(−0.24)
Gender (female)				0.022
				(0.07)
PERIOD	Yes	No	Yes	Yes
INDIVIDUAL	Yes	No	No	No
*R* ^2^	0.05	0.02	0.05	0.05
F	2.691***	9.975***	2.544***	2.117**
*Pr*(*F*>*f*_0_)	0.000	0.002	0.005	0.015
N	528	528	528	528

**Notes**: Dependent variable: difference in the level of the participant's mental stress between the previous and current period. Significance levels: * = 0.05<*P*<0.10, ** = 0.01<*P*<0.05 and *** = *P*<0.01. *t*-statistics in parentheses.

These regression results, although they must be taken with a grain of salt because of the model's very low explained variance, nevertheless preclude outright rejection of the hypothesis that proposers mitigate their stressful experiences by raising their offers. Unfortunately, however, the relatively large heterogeneity of participants – even in the homogeneous student pool – does not allow meaningful further examination, such as out-of-sample predictions. Rather, additional research is needed to assess whether such behaviour actually takes place or whether individuals are in fact able to fully anticipate their emotional reactions. It should also be noted that the manipulations employed in the experiment are quite weak: the stakes for a single game round are reasonably low and social identification with the opposite participant is kept to a low level. Thus, further experiments altering these variables could provide more reliable answers to this question.

## Conclusions

This study uses HRV technology to measure the physiological reactions of participants in a standard economic bargaining experiment. Because the HRV recording device is small and non-intrusive, it allows us to retain the standard setup of an economic experiment, including the interactions between several individuals. The heart rate signal is decomposed using standard time-frequency tools such as the SPWVD wavelet transformation, which allows identification of the ANS activity in several frequency bands related to the activity of different ANS subsystems. The ratio of activity in the high frequency band to that in the low frequency band signals the balance between the sympathetic and parasympathetic systems, which is an indicator of mental stress. We examine the effect of economic decision making on both responders and proposers by conducting an ultimatum bargaining experiment with first-year students at the Queensland University of Technology.

Our results are strongly connected to recent findings in neuroeconomic and neuroscience research, which uses brain-scanning technology to relate activity in different areas of the brain to specific behaviour. Such research shows not only that emotional factors play a key role in determining participants' behaviour in the ultimatum bargaining game [2] but that the emotional responses detected in the anterior cingulate cortex (ACC) are reflected in HRV measurements. Our HRV monitoring equipment can thus indirectly record emotional signals in a far less intrusive manner while also allowing an increased number of experimental participants. Moreover, whereas most neuroeconomics studies focus only on responder's reactions, we are able to explore the behaviour and physiological reactions of both proposers and responders. However, as mentioned in the introduction, HRV provides only an *indirect* measure of neural processes and is only an *index* of regulated emotional responding, unable to take fully into account and understand properly the multifaceted processes of emotions.

The ability to examine the physiological reactions of proposers and responders is a new development in neuroeconomics, although economics has long recognised emotional reactions as a strategy for fostering mutual reciprocity and reputation and punishing uncooperative others [15], [37]. Future studies might therefore consider implementing a dual scanning technique in which two brain scanners record the simultaneous neural responses of two individuals intereacting in a bargaining experiment [38]. The mental stress indicator in our method, however, does allow identification of the post-decision stress levels of both proposers and responders. We find that both the proposer and the responder show stress responses to low offers, with responders experiencing increased stress levels on accepting any offers below 40 percent. Assuming that the mechanism connecting mental stress to physiological arousal is correctly identified, our method may offer one possible way to measure the mental stress induced by economic decision making, as well as allowing identification of the stress component in reaction to changes in relative outcomes.

Nevertheless, although these initial results point to a correlation between decision making and physiological reaction, we find only weak evidence that mitigation of stressful experiences by behavioural adaptation occurs even in the artificial computer lab environment, which raises the question of effect direction. That is, do emotions dictate behaviour or does behaviour induce emotional response? Although our results can give no definite answer to this question, our second set of results clearly indicates some sort of link between the emotional state and the decision. Admittedly, it may be extremely difficult to distinguish the mental processes that determine behaviour from those associated with emotions. Nevertheless, HRV technology is very well-suited to further exploring these issues because the manipulations required, such as establishing stronger social ties between the participants, demand greater flexibility in the experimental setup than other physiological measurement methods can currently provide.

One recent development in neuroscience is an effort to differentiate between positive and negative emotions, particularly as they concern the (ventral) striatum, which is strongly linked to reward, and the amygdala, which is strongly associated with negative emotional processes [39]. For example, Tabibnia, Satpute, and Lieberman [40] find that the ventral striatum, amygdala, ventromedial prefrontal cortex (VMPFC), orbitofrontal cortex (OFC), and a midbrain region near the substantia nigra show greater activity during high fairness offers than during low fairness offers, indicating that individuals react to positive experiences (fairness). This finding raises the question of whether fair offers induce positive emotional responses. According Tabibnia and Lieberman [39], fair offers are linked to higher self-reported happiness and increased activity in various reward regions of the brain. Future studies using non-intrusive methods could thus provide new insights into fairness, happiness, and stress.

Another promising avenue for future research is to increase the possible set of actions to be more consistent with real world experiences. For example, whereas the responders in our experiment expressed their anger and emotions through punishment (rejection), Xiao and Houser [41] introduce an alternative and less expensive emotional outlet – the chance to write a message to the proposer at no pecuniary cost. They find that responders are less likely to use punishment via rejection of unfair offers when they can use this medium to express negative emotions towards the proposer. It is also possible that in addition to experiencing stress when putting forward unfair offers, some proposers may experience pleasure when making fair offers. For instance, after asking questions designed to generate emotional proxies, Haselhuhn and Mellers [42] report that 10 percent of their proposers experienced greater pleasure from fair payoffs than from larger payoffs, an issue that warrants further study using neuroscientific tools. It would also be interesting to determine whether loyalty or positive feedback is linked to emotions. Whatever the goal, the ECG recorder, as well as similarly non-intrusive tools, provides researchers with a valuable opportunity to explore multiple subjects engaged in multiple social interactions, a setting that better approximates real life situations [43].

## Supporting Information

File S1 **This file contains Figure S1-Figure S8 and Table S1.** Figure S1. Holter Medilog Digital ECG Recorder AR4. Figure S2. Instruction Screen for the Ultimatum Bargaining Game. Figure S3. Ultimatum Bargaining Game Test Questions. Figure S4. Ultimatum Bargaining Game Failure to Complete Test Questions. Figure S5. Ultimatum Bargaining Game Offer Screen (Proposer). Figure S6. Ultimatum Bargaining Game Acceptance Screen (Responder). Figure S7. Ultimatum Bargaining Game Summary Screen. Figure S8. Risk Attitudes Elicitation Screen. Table S1. Summary of the Excluded Data.
(DOCX)
