# Supplementary material for: Heartbeat and Economic Decisions: Observing Mental Stress among Proposers and Responders in the Ultimatum Bargaining Game
Source: PLoS One. 2014 Sep 23;9(9):e108218. doi: 10.1371/journal.pone.0108218 (PMC4172752; doi:10.1371/journal.pone.0108218)

SUPPORTING INFORMATION

This study has been approved by the QUT University Human Research Ethics Committee (UHREC, [ethicscontact@qut.edu.au](mailto:ethicscontact@qut.edu.au)). The study involved written informed consent of participants and this procedure was approved by the ethics committee.

*Figure S1*: Holter Medilog Digital ECG Recorder AR4 (Source: schiller.ch)


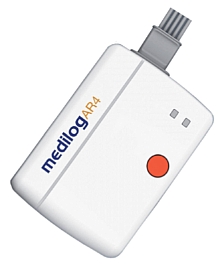


*Figure S2*: Instruction Screen for the Ultimatum Bargaining Game


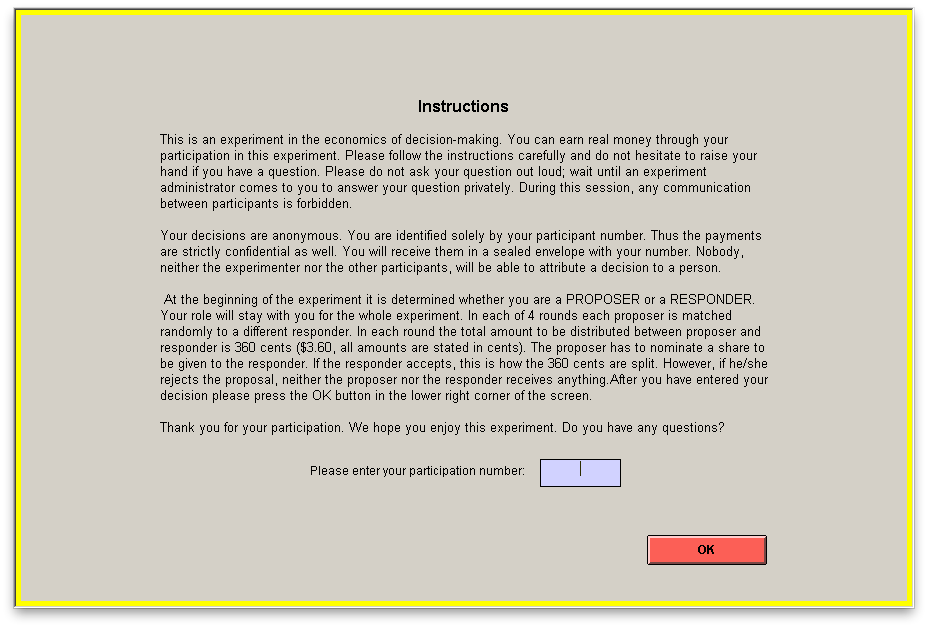


*Figure S3:* Ultimatum Bargaining Game Test Questions


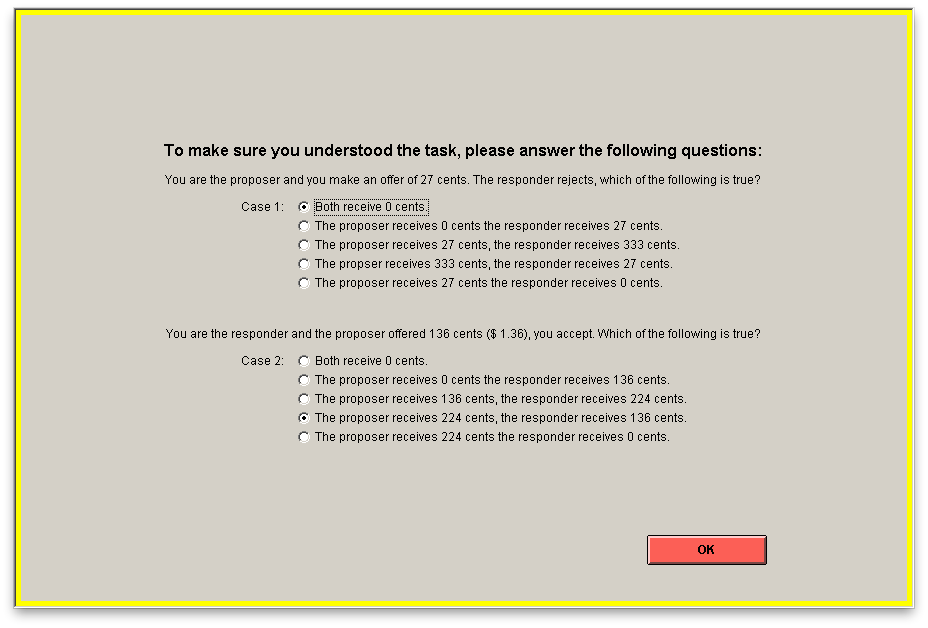


*Figure S4*: Ultimatum Bargaining Game Failure to Complete Test Questions


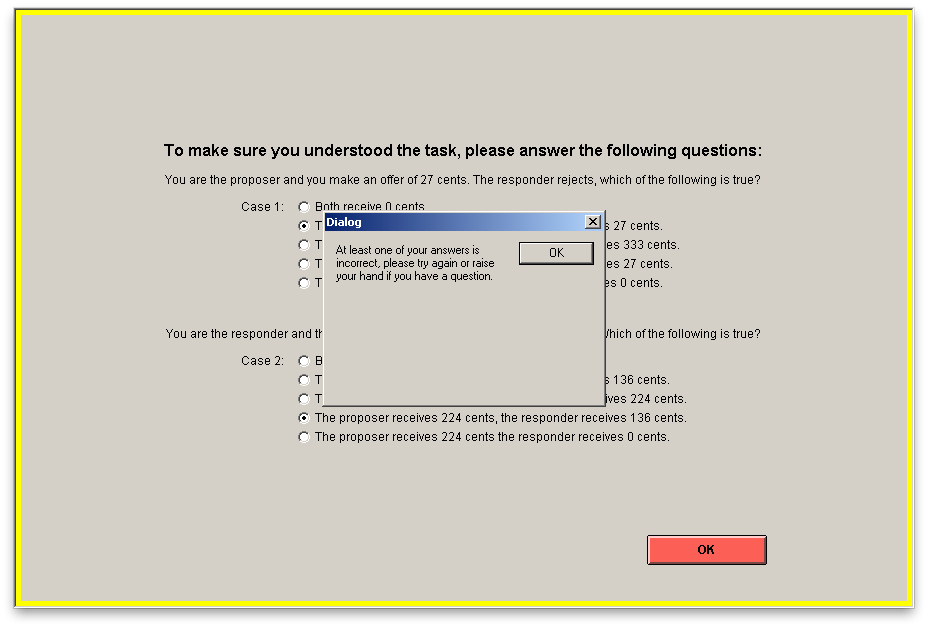


*Figure S5*: Ultimatum Bargaining Game Offer Screen (Proposer).


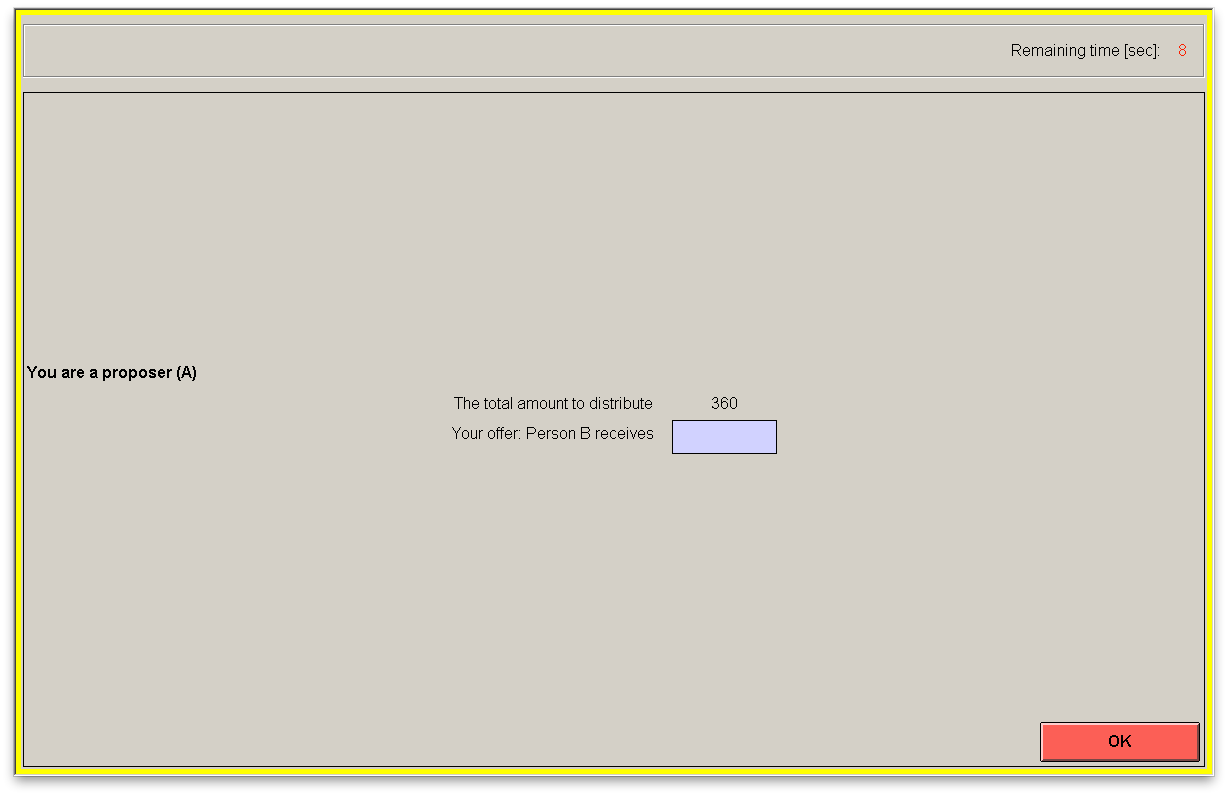


*Figure S6*: Ultimatum Bargaining Game Acceptance Screen (Responder)


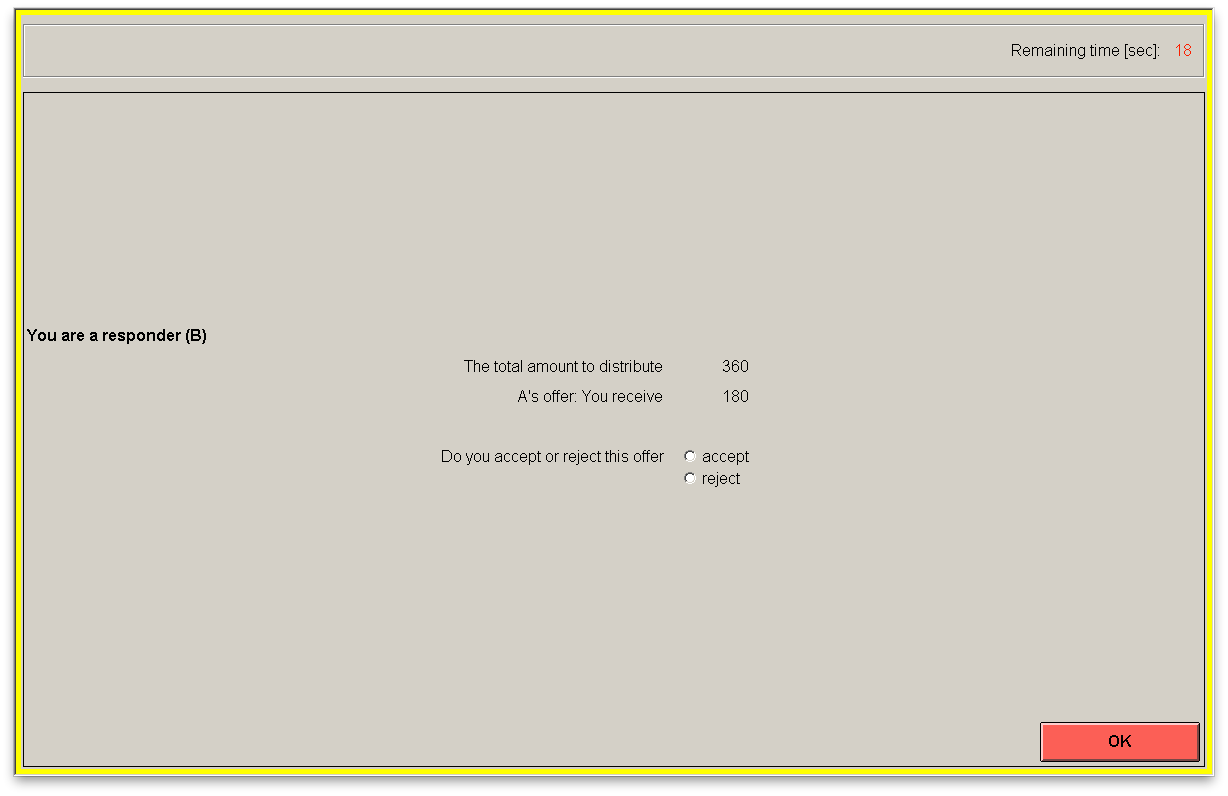


*Figure S7*: Ultimatum Bargaining Game Summary Screen


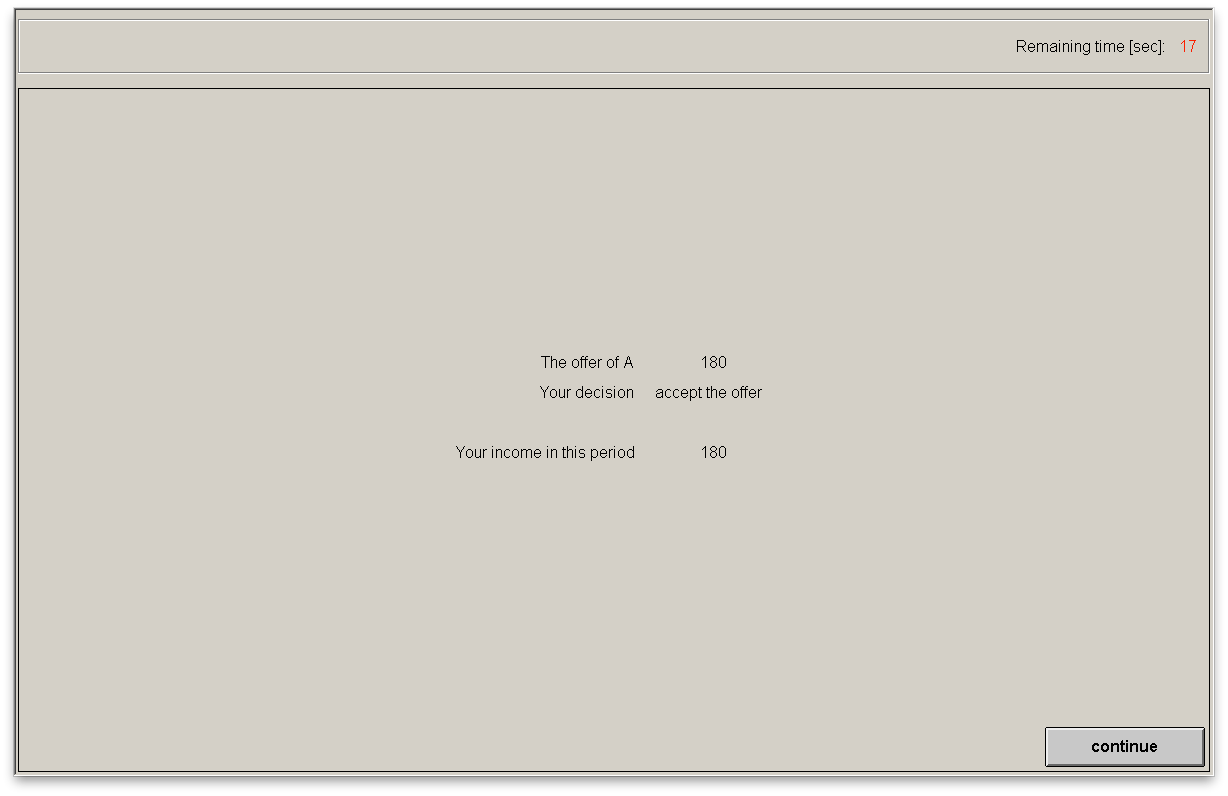


*Table S1*: Summary of the Excluded Data

| **group** | **N** | **offer** | **offer** | **accept** | **accept** | **decl.** | **gender** | **age** |
| --- | --- | --- | --- | --- | --- | --- | --- | --- |
|  |  | cents | % | % | offer % | offer % | female % | years |
| all | 156 | 152.02 | 42.23 | 71.3 | 45.99 | 33.02 | 42.95 | 21.14 |
|  |  | (28.21) | (7.84) | (21.08) | (9.19) | (11.86) | (49.66) | (4.42) |
| included | 130 | 151.62 | 42.38 | 69.21 | 47.58 | 31.88 | 43.85 | 21.38 |
|  |  | (37.32) | (4.61) | (25.86) | (7.63) | (10.36) | (49.81) | (4.54) |
| excluded | 26 | 154.41 | 42.15 | 80.88 | 43.88 | 29.5 | 38.46 | 19.92 |
|  |  | (34.7) | (6.89) | (16.38) | (2.97) | (12.98) | (49.61) | (3.59) |

*Note*: Standard deviations in brackets.

*Figure S8*: Risk Attitudes Elicitation Screen


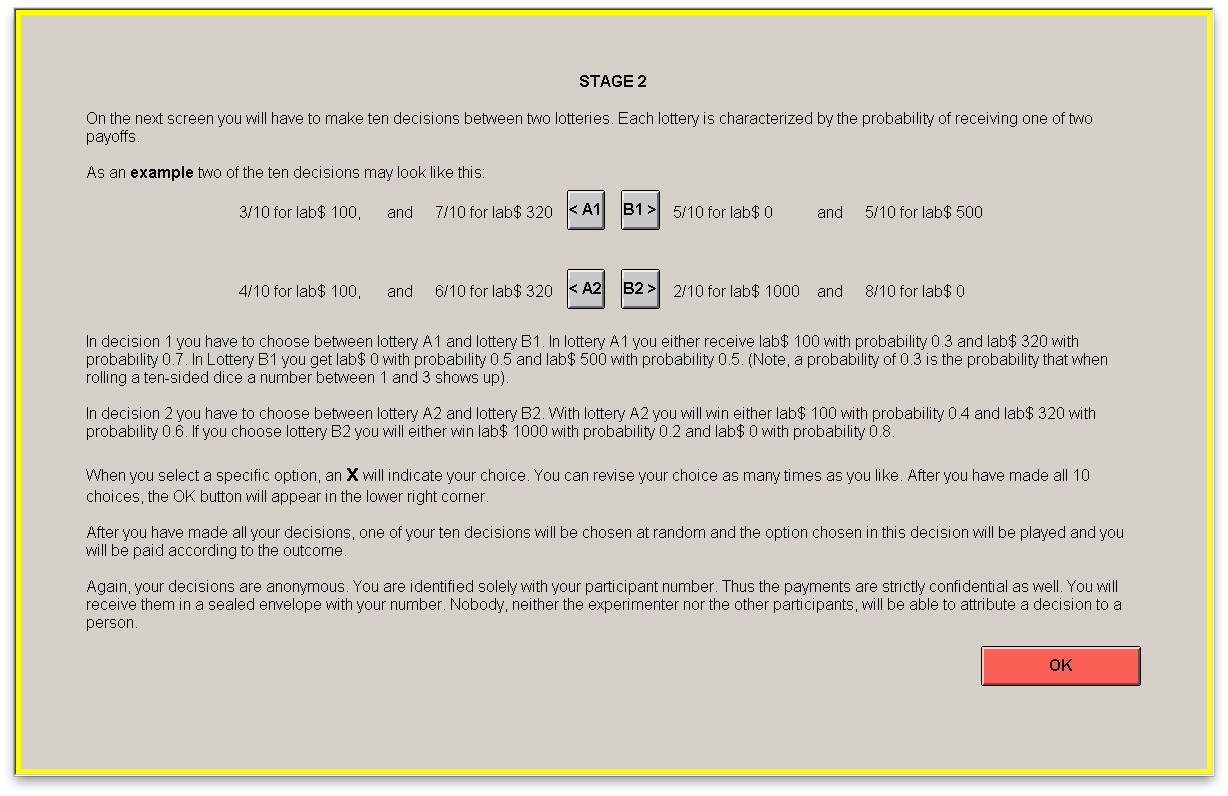

Supplement: File S1 — This file contains Figure S1-Figure S8 and Table S1. Figure S1. Holter Medilog Digital ECG Recorder AR4. Figure S2. Instruction Screen for the Ultimatum Bargaining Game. Figure S3. Ultimatum Bargaining Game Test Questions. Figure S4. Ultimatum Bargaining Game Failure to Complete Test Questions. Figure S5. Ultimatum Bargaining Game Offer Screen (Proposer). Figure S6. Ultimatum Bargaining Game Acceptance Screen (Responder). Figure S7. Ultimatum Bargaining Game Summary Screen. Figure S8. Risk Attitudes Elicitation Screen. Table S1. Summary of the Excluded Data. (DOCX) [file pone.0108218.s001.docx]
